# Absorption of Nutrient Enemata

**Published:** 1914-01

**Authors:** 


Absorption of Nutrient Enemata. A. R. Short and H. W. Bywater, *Brit. Med. Jour.*, June 28, 1913, hold that the method of gauging absorption of enemata by substraction of nutrients remaining in "washouts" is unreliable. Estimations of urinary nitrogen in patients nourished(?) by the rectum, even if the enemata are peptonized for 20—30 minutes, show no appreciable absorption of proteid. This is logical, since modern research supports the theory that proteid is absorbed principally as amino-acids. Using chemically prepared amino-acids or milk peptonized for twenty-four hours, they find a genuine absorption of nitrogen and no marked putrefaction in the bowel contents. Dextrose is well absorbed, better than lactose and sufficiently to relieve acidosis. Fat is scarcely absorbed at all. They boil 750 c.c. of milk, cool, add 15 c.c. of a reliable liquid pancreatic extract or four tablets and incubate at body temperature for twenty-four hours. 150 c.c. every four hours or 300 c.c. every eight hours, are injected.

